# Polo-like kinase 4 and Stromal antigen 3 are not associated with recurrent pregnancy loss caused by embryonic aneuploidy

**DOI:** 10.1038/s41439-020-0106-2

**Published:** 2020-05-29

**Authors:** Hiroyuki Yoshihara, Mayumi Sugiura-Ogasawara, Fumiko Ozawa, Tamao Kitaori, Yasuhiko Ozaki, Koji Aoki, Yasuhiro Shibata, Shinya Ugawa, Takeshi Nishiyama, Yosuke Omae, Katsushi Tokunaga

**Affiliations:** 1 Department of Obstetrics and Gynecology, Nagoya City University, Graduate School of Medical Sciences, Nagoya, Japan; 2 Aoki Ladies Clinic, Graduate School of Medicine, The University of Tokyo, Tokyo, Japan; 3 Anatomy and Neuroscience, Nagoya City University, Graduate School of Medical Sciences, Nagoya, Japan; 4 Public Health, Nagoya City University, Graduate School of Medical Sciences, Nagoya, Japan; 5 Department of Human Genetics, Graduate School of Medicine, The University of Tokyo, Tokyo, Japan

**Keywords:** Genetic association study, Genetics

## Abstract

No genetic association with recurrent pregnancy loss (RPL) caused by embryonic aneuploidy has been found. Recent studies have indicated that the common genetic variant rs2305957, surrounding the *PLK4* gene, contributes to mitotic-origin aneuploidy risk during human early embryo development. The decrease in meiosis-specific cohesin causes predivision of sister chromatids in the centromere and chromosome segregation errors. *STAG3* is a component of cohesin and is a meiosis-specific gene. Our case-control study included 184 patients with RPL whose previous products of conception (POC) exhibited aneuploidy and 190 fertile control women without a history of miscarriage. We performed a genetic association study to examine the genotype distribution at *PLK4* (rs2305957) and *STAG3* in patients with RPL caused by aneuploidy compared with controls. Regarding *STAG3*, SNPs with a minor allele frequency (MAF) threshold > 0.05 that were predicted to be binding sites of transcription factors and that showed significant associations in expression quantitative trait locus (e-QTL) analysis were selected. No significant differences in the MAF or distribution in any model of *PLK4* (rs2305957) and 5 selected tag SNPs in *STAG3* were found between the patients and controls. A further genome-wide association study is needed since a combination of genetic risk alleles might be useful in predicting future age-dependent RPL caused by aneuploidy.

## Pregnancy: Finding genes to predict recurrent pregnancy loss

Researchers have ruled out two potential genetic causes of recurrent pregnancy loss (RPL). Defined as loss of two or more pregnancies at any stage, RPL is caused by loss or gain of a chromosome (aneuploidy) in almost half of cases. Identifying genetic factors could help to predict the risk of RPL, which increases with maternal age. Hiroyuki Yoshihara at Nagoya City University in Japan and co-workers investigated whether two genes involved in chromosome distribution during cell division were associated with aneuploidy-related RPL. In a study of almost 400 women, half of whom had experienced RPL, they showed that neither gene was involved. Because multiple genetic factors could combine to cause RPL, the researchers recommend a genome-wide search. A better understanding of the genetic risk factors of RPL could help women in making fertility-related choices.

## Introduction

Recurrent pregnancy loss (RPL) is defined as two or more losses at any time during pregnancy^1,2^. Most of these occur before 10 weeks of gestation. Identifiable causes of RPL include antiphospholipid syndrome, uterine anomalies, and parental and embryonic chromosomal abnormalities^1,2^. Embryonic or fatal aneuploidy is the most common cause of RPL, with a frequency of 40–50%^3,4^. As maternal age rises, the probability of a newborn baby with aneuploidy increases. The frequency of aneuploidy in embryos and miscarried products of conception (POC) is high in elderly women as well. Oocyte aneuploidy increases as the dictyate arrest is longer due to aging, and the division error occurs mainly in the first division of meiosis.

Sister chromosomes replicated during the DNA synthesis phase (S phase) of the cell cycle are attached by the cohesin protein complex. Between somatic division and meiosis, the components of cohesin are different. Stromal antigen 3 (*STAG3)* is a component of cohesin and is a meiosis-specific gene expressed only in the early embryonic ovary. It has been reported that *Stag3*-deficient mice are sterile, with oocyte formation stopping early in development^5^. In humans, familial ovarian insufficiency can occur through *STAG3* deletion^6^. It is known that no new cohesin is produced in the meiotic arrest phase, and it disappears from the chromosome with advancing age^7^.

On the other hand, Polo-like kinase 4 (*PLK4*) is a unique member of the PLK family that plays vital roles in centriole biogenesis during mitosis. It has been well characterized as the master regulator of centriole duplication, a key component of the centrosome cycle and is essential for mediating bipolar spindle formation during the first cell division in mouse embryos^8–10^. The depletion of maternal *PLK4* prevents nucleation and growth of microtubules and results in abnormal spindle formation, which leads to cytokinesis failure^10^.

Recently, 472 variants in 187 genes have been reported to be associated with unexplained RPL. A meta-analysis revealed a significant association between recurrent miscarriage and 21 genetic variants with ORs of 0.51–2.37^11^. However, genes related to embryonic aneuploidy have not yet been identified. Although *PLK4* is a mitotic-origin aneuploidy-associated gene, there is a report that the rs2305957 polymorphism is related to unexplained recurrent miscarriage^12^. However, the study did not focus on patients whose POC showed aneuploidy.

In the present study, we examined the respective association between RPL and *STAG3* or *PLK4* rs2305957 polymorphism in RPL patients whose previous POC showed aneuploidy.

## Materials and methods

### Study population

We analyzed the data of 184 Japanese patients with a history of two or more unexplained pregnancy losses and at least one miscarriage whose POC showed aneuploidy. All patients were recruited from Nagoya City University Hospital and Aoki Ladies Clinic. All patients underwent a systematic examination, including 4D-ultrasound sonography and/or hysterosalpingography, chromosome analysis of both partners, determination of antiphospholipid antibody, including lupus anticoagulant, diluted activated partial prothrombin time, diluted Russell Russel viper venom time and β2 glycoprotein I-dependent anticardiolipin antibody, and blood tests for hypothyroidism and diabetes mellitus, before a subsequent pregnancy. Patients with antiphospholipid syndrome, an abnormal chromosome in either partner, or uterine anomaly were excluded. Patients whose previously miscarried POC exhibited triploidy or 45,X were excluded.

A total of 190 women with at least one child and no history of miscarriage were examined as control subjects. The control subjects were recruited from Nagoya City University Hospital.

### Ethical approval

This study was conducted with the approval of the Research Ethics Committee of Nagoya City University Graduate School of Medical Sciences. Each patient provided written consent after being given a full explanation of the purpose of the study and the methods to be employed.

### Single-nucleotide polymorphism selection of the genes

**Fig. 1:**
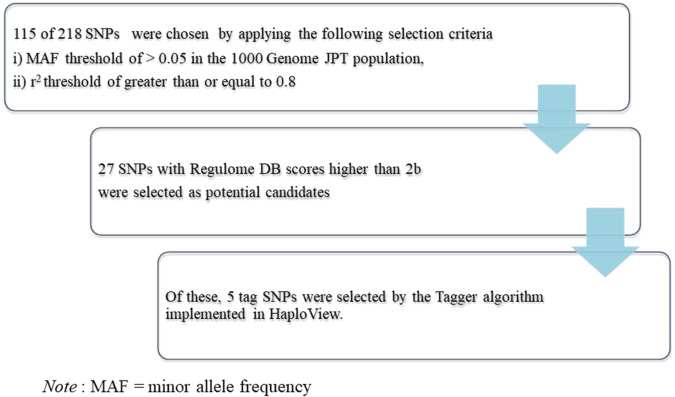
Single-nucleotide polymorphism (SNP) selection of the *STAG3* genes.

We focused on *STAG3* as a meiosis-related gene and *PLK4* as a mitotic-origin aneuploidy risk. Genotype information on 218 *STAG3* Single-nucleotide polymorphisms (SNPs) was downloaded from Phase III of the 1000 Genomes JPT population database (http://browser.1000genomes.org/Homosapiens/UserData/Haploview). The 1000 Genomes data were analyzed using HaploView software (ver4.2). SNPs were chosen by applying the following selection criteria: (i) a minor allele frequency (MAF) threshold of >0.05 in the 1000 Genomes JPT population, (ii) an *r*^2^ threshold of ≥0.8. A total of 115 SNPs meeting the criteria were selected (Fig. 1).

From the 115 SNPs, those that were predicted to be binding sites of transcription factors and those that showed significant associations in e-QTL analysis were selected using the Regulome DB database (http://www.regulomedb.org/index). Next, 27 SNPs with Regulome DB scores higher than 2b (supported by the data of e-QTL and transcription factor binding/DNase peak) were selected as potential candidates. The Regulome DB collects and annotates SNPs with known and predicted regulatory elements in the intergenic regions of the human genome. The RegulomeDB score provides a model integrating functional genomics features along with continuous values such as the ChIP-seq signal, DNase-seq signal, informational content changes, and DeepSEA scores^13^. Of these, 5 tag SNPs were selected by the Tagger algorithm implemented in HaploView, with an additional SNP reported previously, to validate their association with miscarriages with an abnormal number of embryonic chromosomes.

### DNA analysis

Genomic DNA was extracted from peripheral blood samples using the Midi Blood DNA Extraction Kit (QIAGEN).

All genotyping was carried out using TaqMan polymerase chain reaction (PCR) assays (Applied Biosystems) in 96-well arrays that included blank wells as negative controls according to the manufacturer’s instructions. TaqMan Predesigned SNP Genotyping and TaqMan MGB probes were used. TaqMan PCR and genotyping analyses were carried out on the Applied Biosystems 7500 Fast Real-Time PCR system. The reaction mixtures consisted of 2 µL template DNA (5 ng/µL), 5 µL 2×TaqMan GTXpress Master Mix, 0.25 µL 40×primer/probe mix, and 2.75 µL double-distilled H_2_O in a 10 µL volume. The cycling conditions were as follows: initial denaturation at 95 °C for 20 s, followed by 40 cycles at 95 °C for 3 s and then 60 °C for 30 s. The results were automatically analyzed on the Applied Biosystems 7500 Real-Time PCR system with an allelic discrimination assay program^14^.

### Statistical analysis

Departure from Hardy–Weinberg equilibrium for the six SNPs was determined using the exact test^15^. Because previous studies have shown that the mainland Japanese population is genetically similar, we did not examine or perform corrections for the population substructure of our sample^16,17^.

For association analyses of individual SNPs, univariate logistic regression analyses were performed using the presence or absence of RPL as the dependent variable. There remains some uncertainty about the underlying genetic model. Therefore, to examine the right model of inheritance statistically as well as to avoid multiple comparisons by fitting multiple inheritance models, we used the max-statistic, which selects the largest test statistic from the dominant, recessive, and additive models, using the SNPassoc package^18^. Since the AA genotype of rs941288 was not observed in the sample, we conducted Fisher’s exact test for the SNP instead of the max-test.

To characterize the linkage disequilibrium (LD) pattern, we estimated the *r*^2^ values for all pairs of SNPs. Haplotype analysis was conducted within blocks of LD as defined *r*^2^ ≥ 0.6, using the R haplo.stats package^19^. The association of common haplotypes with the risk of recurrent pregnancy loss was estimated with the R function haplo.glm. Haplo.glm applies a haplotype-trait association test based on a general linear model framework using maximum likelihood estimates for haplotype effects, allowing for ambiguity of the haplotype phase. A log-additive risk model was assumed, in which haplotype-specific regression coefficients represent the change in the log odds of disease for every additional copy of the haplotype compared with the homozygote reference haplotype.

The max-test is similar to or marginally better in power than the chi-square test based on the codominant model^20^. Therefore, the power analysis was carried out based on the codominant model using CaTS^21^. Given our sample size, our study has 60–80% power to detect a genotype risk ratio of 1.5 under the multiplicative model when the allele frequency is within the range observed in the sample (20–50%) (Supplementary Fig.). Note that the power estimate based on the codominant model is slightly conservative for the max-test.

All statistical analyses were conducted with R software (v.3.6.1)^22^. *P* < 0.05 was considered statistically significant.

## Results

Characteristics of RPL patients and control subjects are presented in Table 1. The mean (SD) ages of the patients and control subjects were 36.8 (4.3) and 36.8 (5.5) years, respectively. The mean (SD) number of previous miscarriages was 3.09 (1.13).

**Table 1:** Characteristics of patients with RPL whose previous aborted conceptus was ascertained to exhibit aneuploidy.

	Patients	Control	*P*-value
Number of patients	184	190	
Mean age (SD)	36.8 ± 4.3	36.8 ± 5.5	0.92
Number of previous miscarriages	3.09 ± 1.13	0	<0.0001
Number of previous live births	0.46 ± 0.64	1.66 ± 0.70	<0.0001
Previous IVF-ET	26.1% (48)	–	–

*RPL* recurrent pregnancy loss, *IVF-ET* in vitro fertilization-embryo transfer.

Values are mean ± SD or *n* (%) except for number of patients.

A total of six SNPs were analyzed: rs2305957 (G>A at *PLK4*;SNP1), rs941288 (G>A at *STAG3*;SNP2), rs13230744 (G>A at *STAG3*;SNP3), rs1061230 (G>A at *STAG3*;SNP4), rs1624099 (G>A at *STAG3*;SNP5), and rs4727450 (G>A at *STAG3*;SNP6).

The genotype frequencies for the six SNPs were found to be in Hardy-Weinberg equilibrium, suggesting that there was neither sampling bias nor mistyping in genotyping. The MAF of *PLK4* (rs2305957) was 0.367 for patients and 0.361 for controls (Table 2), similar to that in the Japanese population. The MAF(A) of that in the JPT was reported to be 0.365 in the Integrative Japanese Genome Variation Database. Based on the results of the max-statistics, the recessive model was adopted. No significant difference in the distribution of the recessive model of rs2305957 (G>A) was found between the patients and controls (Table 3).

**Table 2:** Genotype allele frequencies of PLK4 and STAG3 polymorphisms among RPL patients and controls.

	Patients *n* = 184 (%)	Controls *n* = 190 (%)	HWE
SNP1 *PLK4* (rs2305957)			
GG	78 (42.4)	77 (40.5)	0.435
GA	77 (41.8)	89 (46.8)	
AA	29 (15.8)	24 (12.6)	
G	233 (63.3)	243 (63.9)	
A	135 (36.7)	137 (36.1)	
SNP2 *STAG3* (rs941288)			
GG	155 (84.2)	170 (89.5)	0.388
GA	29 (15.8)	20 (10.5)	
AA	0 (0)	0 (0)	
G	339 (92.1)	360 (94.7)	
A	29 (7.9)	20 (5.3)	
SNP3 *STAG3* (rs13230744)			
GG	71 (38.6)	78 (41.1)	0.324
GA	81 (44.0)	86 (45.3)	
AA	32 (17.4)	26 (13.7)	
G	223 (60.6)	242 (63.7)	
A	145 (39.4)	138 (36.3)	
SNP4 *STAG3* (rs1061230)			
GG	59 (32.1)	66 (34.7)	0.752
GA	88 (47.8)	92 (48.4)	
AA	37 (20.1)	32 (16.8)	
G	206 (56.0)	224 (58.9)	
A	162 (44.0)	156 (41.1)	
SNP5 *STAG3* (rs1624099)			
GG	77 (41.8)	77 (40.5)	1.000
GA	82 (44.6)	91 (47.9)	
AA	25 (13.6)	22 (11.6)	
G	236 (64.1)	245 (64.5)	
A	132 (35.9)	135 (35.5)	
SNP6 *STAG3* (rs4727450)			
GG	107 (58.2)	106 (55.8)	0.273
GA	64 (34.8)	69 (36.3)	
AA	13 (7.1)	15 (7.9)	
G	278 (75.5)	281 (73.9)	
A	90 (24.5)	99 (26.1)	

*HWE* Hardy–Weinberg equilibrium.

**Table 3:** Results of maximizing association statistics.

	SNP1	SNP3	SNP4	SNP5	SNP6
Dominant	0.13	0.24	0.30	0.07	0.21
Recessive	0.75	0.98	0.66	0.34	0.09
Log-additive	0.03	0.72	0.66	0.01	0.24
Max-statistic	0.75	0.98	0.66	0.34	0.24
*P* value	0.619	0.537	0.655	0.803	0.857

Associations are assessed using maximized *χ*^2^ statistics, which select the largest test statistics from the dominant, recessive, and additive models.

*SNP1* rs2305957, *SNP2* rs941288, *SNP3* rs13230744, *SNP4* 1061230, *SNP5* rs1624099, *SNP6* rs4727450.

The MAF of *STAG3* (rs941288) was 0.079 for patients and 0.053 for controls. The MAF of *STAG3* (rs13230744) was 0.364 for patients and 0.393 for controls. The MAF of *STAG3* (rs1061230) was 0.440 for patients and 0.411 for controls. The MAF of *STAG3* (rs1624099) was 0.359 for patients and 0.374 for controls. The MAF of *STAG3* (rs4727450) was 0.245 for patients and 0.261 for controls. Since the AA genotype of rs941288 was not observed in the sample, we conducted Fisher’s exact test for the SNP and found no association with RPL (*p*-value = 0.168). For the remaining five SNPs, associations were assessed using maximized *χ*^2^ statistics, where selections corresponded to the largest test statistics from the dominant, recessive and additive models.

**Fig. 2:**
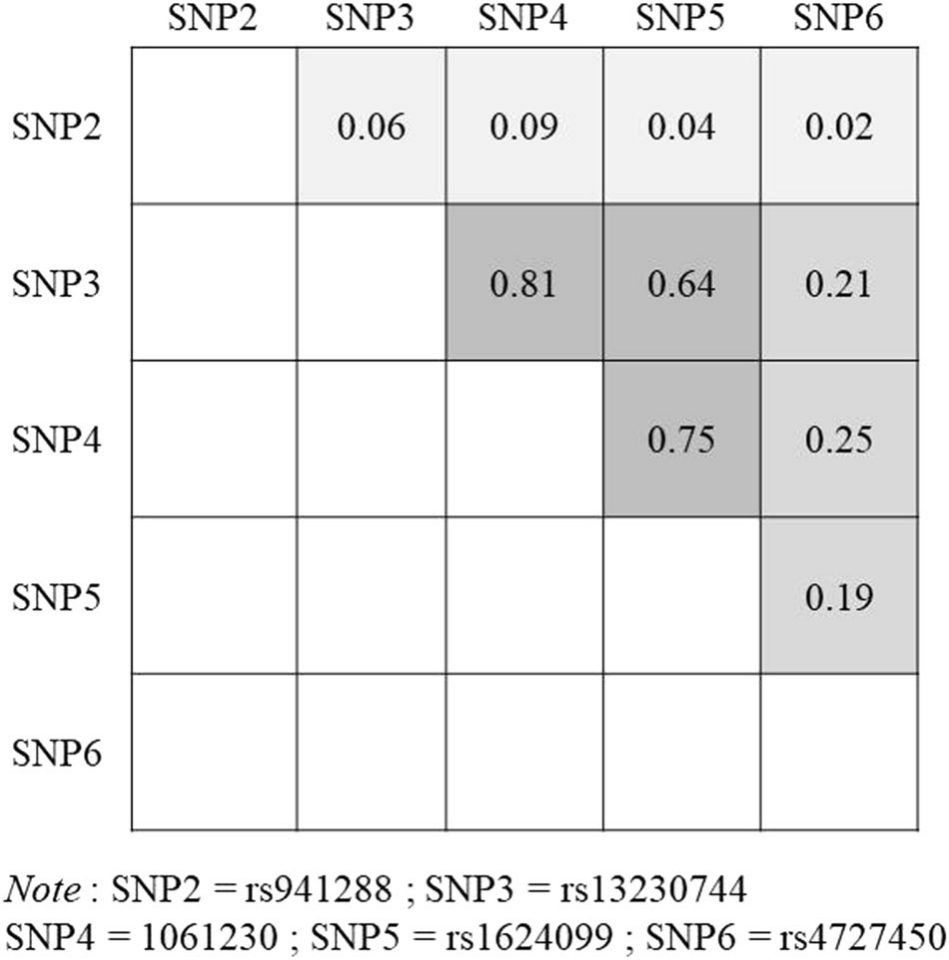
Linkage disequilibrium (*r*^2^ value) of the five *STAG3* single-nucleotide polymorphism.

The results of the max-statistics are shown for all five polymorphisms (Table 3), and they suggested no statistically significant associations with any of the five SNPs. We also performed an LD analysis of the five *STAG3* SNPs (Fig. 2), which revealed an LD block among SNPs 3, 4, and 5. Analysis of the region within the LD block indicated the presence of four major haplotypes. G-G-G (57.3%), A-A-A (32.1%), A-A-G (5.56%), and G-A-A (3.56%) comprised the major haplotypes and accounted for > 98% of the subjects, and no haplotype was significantly associated with an RPL risk compared with the most common haplotype, G-G-G, under the log-additive model (Table 4).

**Table 4:** Results of haplotype analysis.

Haplotype	SNP3	SNP4	SNP5	Frequency	*P*-value	OR (95% CI)
A-A-A	A	A	A	0.321	0.745	1.05 (0.77–1.44)
A-A-G	A	A	G	0.056	0.085	1.82 (0.92–3.60)
G-A-A	G	A	A	0.036	0.984	1.01 (0.44–2.30)
Others	^a^	^a^	^a^	0.014	0.644	0.73 (0.19–2.78)
G-G-G	G	G	G	0.573	–	Reference

*SNP3* rs13230744, *SNP4* 1061230, *SNP5* rs1624099.

^a^means either A or G.

## Discussion

In the present study, we found no significant differences in MAF or any of the genetic models of the 6 SNPs of *PLK4* and *STAG3* between the RPL patients and controls.

We believe that the findings of the present study, where the *PLK4* rs2305957 polymorphism was not associated with RPL, are important. McCoy et al.^23^ examined day 3 blastomeres and blastocysts used for preimplantation genetic testing for aneuploidy and found an association between *PLK4* rs2305957 and maternal mitotic-origin aneuploidy in a genome-wide association study. The study did not find a gene associated with meiotic-origin aneuploidy, and these mitotic errors produce mosaic embryos with two or more cell lineages, each possessing distinct chromosomal complements. Women with the genotypes contributed fewer blastocysts, suggesting that their embryos were less likely to survive blastocyst formation. In a study of 2015 infertile women, Zhang et al.^12^ demonstrated that women with an AA genotype showed significantly lower blastocyst formation and that the variant rs2305957 might represent a maternal risk factor for RPL. The MAF(A) of RPL was reported to be 0.489 and that of controls was 0.425. However, no significant difference in early miscarriage rates or live birth rates was observed among women with AA, AG, and GG genotypes who underwent the standard IVF protocol^12^.

In contrast, we found no association between maternal rs2305957 spanning *PLK4* and RPL. We focused on RPL patients whose POC showed autosomal aneuploidy. Almost all were trisomies. Early studies demonstrated that most aneuploidies in miscarriages and affected children are due to errors in maternal meiosis and that increasing maternal age is a strong contributor to the occurrence of aneuploidy^24^. It was speculated that the risk allele of *PLK4* might contribute not to miscarriage but rather to mosaicism and implantation failure because it was a mitotic-origin aneuploidy gene.

Thus, we focused on a meiosis-origin aneuploidy gene, *STAG3*. In the present study, we focused on the fact that cohesin, which controls chromosome adhesion, decreases with age and performed polymorphism analysis of genes that encode it. Chromosomes replicate in meiosis, and sister chromosomes attach to each other through the action of this protein complex^25^. Cohesin in the centromere is not decomposed, and the spindle microtubule recruits the centromere of the sister chromosome and distributes it to the opposite station. In the second division of meiosis, the remaining cohesin is broken down by separase, and the sister chromosomes are distributed to the opposite poles. Cohesin acts to protect the adhesion of sister chromosomes and the mono-orientation of kinetochores. It is known that no new cohesin is produced in the meiotic arrest phase, and it eventually disappears from the chromosome in an age-dependent manner^7^. We examined *STAG3* since it encodes cohesin and is a meiosis-specific gene expressed only in the early embryonic ovary. However, we failed to show any association with RPL.

*REC8* (meiotic recombination protein 8) forms a synaptonemal complex, promotes recombination, recruits Shugosin and *MEIKIN* protein to protect centromere adhesion and maintains mono-orientation of the centromere structure^26,27^. *MEIKIN* (meiosis-specific kinetochore protein) is specifically expressed and localized in the first division of meiosis in mouse germ cells, and in an analysis of knockout mice, meikin was found to inhibit mono-orientation binding of the centromere and to play an important role in centromere adhesion^28^. One limitation of the present study was that we did not analyze meiosis-associated genes except for *STAG3*. Even if we narrow down gene polymorphisms acquired from a database, the number of polymorphisms with functional alterations is relatively large and unrealistic for practical use. In addition, relatively recently discovered genes have many unreported polymorphisms, making it difficult to conduct a database-dependent analysis.

We could not find any association between *PLK4* or *STAG3* and RPL. A low level of anti-Müllerian hormone can indicate a poorly responsive ovarian follicle but cannot predict the future capability of a live birth. In contrast, a combination of genetic risk alleles might be useful for predicting future age-dependent infertility as well as RPL caused by aneuploidy. A greater knowledge of the collective risk of these alleles may cause women to think carefully about their life plan and encourage them to consider getting pregnant at a younger age. Such information could be highly useful. With this in mind, a genome-wide association study should be undertaken in the near future, as it will benefit not only women in Japan but also those elsewhere.

## Supplementary information

Supplementary Figure

## References

[1] European Society for Human Reproduction and Embryology (ESHRE) early pregnancy guideline development group. Recurrent pregnancy loss guideline of the ESHRE (2017).

[2] Medicine PCoASfR. (2013). Definitions of infertility and recurrent pregnancy loss: a committee opinion. Fertil. Steril..

[3] Sugiura-Ogasawara M (2012). Abnormal embryonic karyotype is the most frequent cause of recurrent miscarriage. Hum. Reprod..

[4] Popescu F, Jaslow CR, Kutteh WH (2018). Recurrent pregnancy loss evaluation combined with 24-chromosome microarray of miscarriage tissue provides a probable or definite cause of pregnancy loss in over 90% of patients. Hum. Reprod..

[5] Prieto I (2001). Mammalian STAG3 is a cohesin specific to sister chromatid arms in meiosis I. Nat. Cell Biol..

[6] Caburet S (2014). Mutant cohesin in premature ovarian failure. N. Engl. J. Med..

[7] Tsutsumi M (2014). Age-related decrease of meiotic cohesins in human oocytes. PLoS ONE.

[8] Habedanck R, Stierhof YD, Wilkinson CJ, Nigg EA (2005). The Polo kinase Plk4 functions in centriole duplication. Nat. Cell Biol..

[9] Bettencourt-Dias M (2005). SAK/PLK4 is required for centriole duplication and flagella development. Curr. Biol..

[10] Coelho PA (2013). Spindle formation in the mouse embryo requires Plk4 in the absence of centrioles. Dev. Cell.

[11] Pereza N, Ostojić S, Kapović M, Peterlin B (2017). Systematic review and meta-analysis of genetic association studies in idiopathic recurrent spontaneous abortion. Fertil. Steril..

[12] Zhang Q (2017). Maternal common variant rs2305957 spanning PLK4 is associated with blastocyst formation and early recurrent miscarriage. Fertil. Steril..

[13] Boyle AP (2012). Annotation of functional variation in personal genomes using RegulomeDB. Genome Res..

[14] Endo K (2005). Epidermal growth factor receptor gene mutation in non-small cell lung cancer using highly sensitive and fast TaqMan PCR assay. Lung Cancer.

[15] Wigginton JE, Cutler DJ, Abecasis GR (2005). A note on exact tests of Hardy-Weinberg equilibrium. Am. J. Hum. Genet..

[16] Nishiyama T (2012). Detailed analysis of Japanese population substructure with a focus on the southwest islands of Japan. PLoS ONE.

[17] Yamaguchi-Kabata Y (2008). Japanese population structure, based on SNP genotypes from 7003 individuals compared to other ethnic groups: effects on population-based association studies. Am. J. Hum. Genet..

[18] González JR (2007). SNPassoc: an R package to perform whole genome association studies. Bioinformatics.

[19] Lake SL (2003). Estimation and tests of haplotype-environment interaction when linkage phase is ambiguous. Hum. Hered..

[20] González JR (2008). Maximizing association statistics over genetic models. Genet. Epidemiol..

[21] Skol AD, Scott LJ, Abecasis GR, Boehnke M (2006). Joint analysis is more efficient than replication-based analysis for two-stage genome-wide association studies. Nat. Genet..

[22] R Development Core Team. *R: A Language And Environment For Statistical Computing* (R Foundation for Statistical Computing, Vienna, Austria, 2011).

[23] McCoy RC (2015). Common variants spanning PLK4 are associated with mitotic-origin aneuploidy in human embryos. Science.

[24] Hassold T, Hunt P (2001). To err (meiotically) is human: the genesis of human aneuploidy. Nat. Rev. Genet..

[25] Watanabe Y (2012). Geometry and force behind kinetochore orientation: lessons from meiosis. Nat. Rev. Mol. Cell Biol..

[26] Kim KP (2010). Sister cohesion and structural axis components mediate homolog bias of meiotic recombination. Cell.

[27] Ishiguro T, Tanaka K, Sakuno T, Watanabe Y (2010). Shugoshin-PP2A counteracts casein-kinase-1-dependent cleavage of Rec8 by separase. Nat. Cell Biol..

[28] Kim J (2015). Meikin is a conserved regulator of meiosis-I-specific kinetochore function. Nature.

