# Supplementary figures and images for: Polo-like kinase 4 and Stromal antigen 3 are not associated with recurrent pregnancy loss caused by embryonic aneuploidy
Source: Hum Genome Var. 2020 May 29;7:18. doi: 10.1038/s41439-020-0106-2 (PMC7260232; doi:10.1038/s41439-020-0106-2)

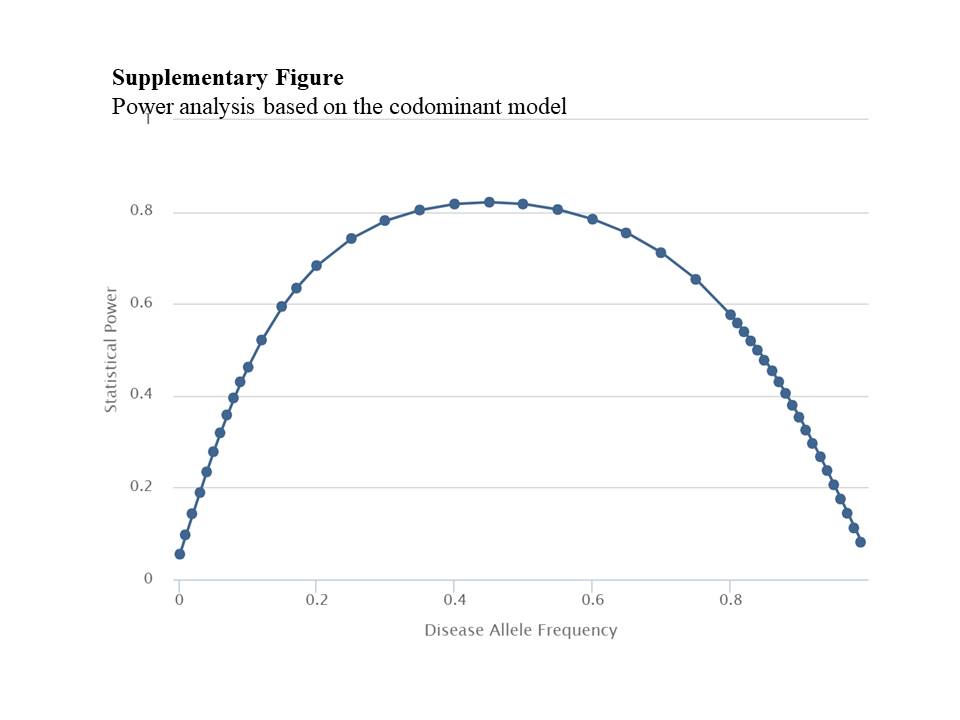

Supplement: Supplementary file 1 — Supplementary Figure [file 41439_2020_106_MOESM1_ESM.jpg]
